# Bacterial community structure in the rumen and hindgut is associated with nitrogen efficiency in Holstein cows

**DOI:** 10.1038/s41598-023-37891-7

**Published:** 2023-07-03

**Authors:** V. M. De La Guardia-Hidrogo, H. A. Paz

**Affiliations:** 1 Department of Animal and Dairy Sciences, Mississippi State University, Mississippi State, MS USA; 2 Department of Pediatrics, College of Medicine, University of Arkansas for Medical Sciences, Little Rock, AR 72205 USA

**Keywords:** Microbial communities, Microbiome, Gastrointestinal system, Microbiota

## Abstract

Nitrogen efficiency (Neff; milk N/N intake) in dairy cows is limited and most of the consumed N is excreted in manure. Despite the crucial role of the gastrointestinal microbiome on N metabolism, associations between bacterial communities at different sections and Neff are not fully elucidated. Enhanced understanding of host-microbiome interactions can provide insights to improve Neff in dairy cows. Twenty-three Holstein cows were selected, and their Neff were determined using a N balance approach. From the cohort of cows, six cows were classified as low Neff and five cows as high Neff and their rumen and fecal bacterial communities were profiled using amplicon sequence variants (ASV) based on 16S rRNA gene sequencing. Then, relationships between differentially abundant bacterial features and Neff were evaluated. Neff in low and high cows averaged 22.8 and 30.3%, respectively. With similar N intake, high Neff cows wasted less N in manure compared to low Neff cows (*P* < 0.01, 11.0 ± 0.59 vs 14.3 ± 0.54 g of N/kg of milk). Rumen fermentation and plasma profiles were similar between Neff groups, but for plasma Gln which was greater (*P* = 0.02) in high compared to low Neff cows. In both rumen and feces, the phylogenetic composition of the bacterial communities was similar (*P* ≥ 0.65) between Neff groups, but differences were observed at the species -level (amplicon sequence variants). In the rumen, differentially abundant species from the genus *Prevotella* showed strong positive correlations with Neff, whereas in feces, differentially abundant species from the class *Clostridia* showed strong negative correlations with Neff. Our results revealed that Holstein cows with divergent Neff display distinctive bacterial community structure at the species-level in both the rumen and feces. Strong correlations between differentially abundant species and Neff in both sample sites, support the importance of the rumen bacterial composition on productive responses and suggest a more relevant role of the hindgut microbiome. Targeting both pre- and post-gastric bacterial communities may provide novel opportunities to enhance Neff in dairy cows.

## Introduction

Dairy cow enterprises are important contributors to global food security supplying ~ 80% of the total milk^1^. Over the past decades, intensification of the dairy sector has resulted in fewer dairy farms with a greater herd size and milk output^2^. Scale-up of dairy operations decreases the cost of milk production, but also results in challenges with nutrient management^3^. Generally, dairy cows exhibit a low N efficiency (Neff; milk N/N intake) and  ~ three-fourths of the consumed N is excreted in manure which is of environmental concern^4,5^. Feed is the greatest cost of milk production and protein supplements are main components in dairy diets, thus improvement in Neff will assist farm finance and reduce N waste^6,7^. Reported Neff of 35% under commercial settings with no adverse impacts on milk yield and composition indicates an opportunity for improvement^8,9^.

The gastrointestinal microbiota serves as an intermediary between dietary inputs and animal end products. In ruminants, the relevance of the rumen microbiota in N metabolism is well recognized as a main portion of the consumed protein is degraded by rumen microbes and microbial crude protein (CP) represents more than 50% of the metabolized protein^10,11^. However, only until recently has the structure of the rumen microbial community gained greater notice. Studies have identified subsets of rumen microbes that can predict productive traits in both dairy and beef cattle^12,13^. These results suggest that specific microbial members can shift rumen fermentation to yield compounds that are of more benefit to the animal. For instance, repeated inoculation with bison rumen contents improved the total tract N digestibility of heifers fed a barley straw diet and this response was correlated with shifts in the bacterial families *Christensenellaceae* and *BS11 gut group* and total protozoa^14^. The microbial community of the hindgut also has the capacity to degrade N sources and plays a role in N recycling^15^. Similar to the rumen, associations between the hindgut microbiome and feed efficiency and milk traits have been reported^16,17^. Evaluations of the relationships between the microbial communities of the rumen and hindgut and Neff are needed to provide insights about host-microbiome interactions to improve the conversion of N into high quality milk protein.

The objectives of this study were to characterize the rumen and fecal bacterial communities of dairy cows with divergent Neff, identify bacterial features that differed between Neff groups, and evaluate the extent of the relationships between the identified differential bacterial features and Neff. We hypothesized that for both the rumen and hindgut, the global profile of the bacterial community will be similar between divergent Neff groups, but differences will be observed for specific groups, and these will show strong correlations with Neff.

## Results

### N efficiency phenotype

Based on the Neff phenotype selection criteria, 6 cows were classified as low Neff and 5 cows were classified as high Neff (Supplementary Table S1). In these cows, Neff ranged from 20.7 to 34.1% and significantly differed (*P* < 0.01; Table 1) between the low and high phenotypes averaging 22.8 and 30.3%, respectively.

**Table 1:** Dry matter intake, milk yield, and apparent nitrogen balance in low and high nitrogen efficient Holstein cows.

Item^1^	Phenotype		*P*-value
	Low Neff (n = 6)	High Neff (n = 5)	
DMI, kg/day	27.8 ± 1.24	25.7 ± 1.36	0.27
Milk yield, kg/day	32.8 ± 1.36	39.4 ± 1.49	< 0.01
Protein, %	3.11 ± 0.08	2.98 ± 0.08	0.27
Fat, %	3.99 ± 0.17	3.68 ± 0.19	0.26
SCC, cells/mL	53.5 ± 2.81	62.6 ± 3.08	0.06
N balance, g/day			
N intake	713 ± 31.6	658 ± 34.6	0.27
Milk N	162 ± 9.65	199 ± 10.57	0.03
Urinary N	222 ± 15.1	212 ± 16.6	0.68
Fecal N	245 ± 12.9	219 ± 14.1	0.20
Manure N	467 ± 21.7	431 ± 23.8	0.29
Retained N	83.1 ± 23.4	27.9 ± 25.7	0.15
N, g of N/kg of milk			
Urinary N	6.76 ± 0.39	5.39 ± 0.42	0.04
Fecal N	7.54 ± 0.36	5.56 ± 0.40	< 0.01
Manure N	14.3 ± 0.54	11.0 ± 0.59	< 0.01
Neff, %	22.8 ± 0.83	30.3 ± 0.91	< 0.01

^1^Values expressed as mean ± SEM.

DMI dry matter intake, SCC somatic cell count

Manure N = urinary N + fecal N.

Retained N = $${\text{N}}\;{\text{intake }} - ({\text{milk}}\;{\text{N}} + {\text{manure }}\;{\text{N}})$$.

N efficiency (Neff) = $$({\text{milk }}\;{\text{N}}\;({\text{g/d}}){\text{/N}}\;{\text{intake}} \;({\text{g/d}})) \times 100$$.

### N balance in low and high Neff cows

Production and N partitioning responses between low and high Neff cows are shown in Table 1. The DMI were similar (*P* = 0.27) between the low and high Neff cows and averaged 26.7 kg/day, but high Neff cows produced 6.6 kg/day more (*P* < 0.01) milk than low Neff cows. Between Neff groups, milk composition did not differ (*P* ≥ 0.26) and averaged 3.00 and 3.84% for protein and fat; respectively, and SCC was also similar (*P* = 0.06) and averaged 58.1 cells/mL. In line with DMI and milk yield responses, N intake was similar (*P* = 0.27) between Neff groups, but milk N, determined from true protein, was greater (*P* = 0.03) in high Neff compared to low Neff cows (199 vs 162 g/day, respectively). Partitioning of N in urine and feces was similar (*P* ≥ 0.20) between Neff groups and consequently manure N (urinary N + fecal N) did not differ (*P* = 0.29). However, when manure N was expressed per kg of milk produced, low Neff cows wasted more N in urine (*P* = 0.04) and feces (*P* < 0.01) than high Neff cows, thus manure N was also greater (*P* < 0.01; 14.3 vs 11.0 g of N/kg of milk, respectively).

### Ruminal fermentation

Ruminal fermentation variables are summarized in Table 2. The rumen environments were comparable between the low and high Neff cows. No differences were observed in pH (*P* = 0.63), NH_3_ concentration (*P* = 0.37), and total VFA concentration (*P* = 0.88). In addition, the molar proportions of acetic (*P* = 0.34), propionic (*P* = 0.35), butyric (*P* = 0.99), and valeric acid (*P* = 0.33) were similar between Neff groups.

**Table 2:** Ruminal fermentation profile in low and high nitrogen efficient Holstein cows.

Item^1^	Phenotype		*P*-value
	Low Neff (n = 6)	High Neff (n = 5)	
Rumen pH	6.56 ± 0.09	6.49 ± 0.10	0.63
Ammonia, mg/dL	8.05 ± 1.34	9.92 ± 1.47	0.37
Total VFA, m*M*	142 ± 14.2	145 ± 15.5	0.88
VFA, mol/100 mol			
Acetic acid	68.4 ± 1.07	66.8 ± 1.17	0.34
Propionic acid	21.5 ± 1.22	23.3 ± 1.34	0.35
Butyric acid	8.96 ± 0.26	8.95 ± 0.29	0.99
Valeric acid	1.12 ± 0.11	0.95 ± 0.11	0.33

Neff N efficiency.

^1^Values expressed as mean ± SEM.

### Plasma AA profile

**Figure 1:**
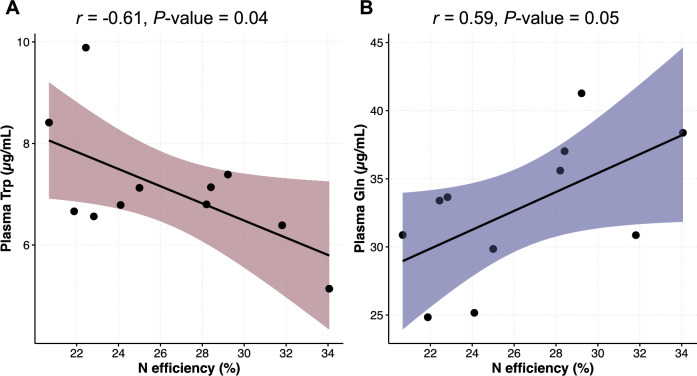
Correlations between plasma (**A**) Trp and (**B**) Gln and nitrogen efficiency in Holstein cows.

Plasma concentration of essential AA (EAA; Arg, His, Ile, Leu, Lys, Met, Phe, Thr, Trp, Val) did not differ (*P* ≥ 0.06) between Neff groups (Supplementary Table S2). Likewise, plasma concentration of total EAA (TEAA) did not differ (*P* = 0.14) and averaged 142 µg/mL. For non-essential AA (NEAA), plasma concentrations between Neff groups were similar (*P* ≥ 0.15) but for Gln (*P* = 0.02) which was greater in high Neff compared to low Neff cows (36.6 vs 29.6 mg/mL). To further evaluate relationships between plasma concentration of individual AA and Neff, Pearson correlations were determined. A moderate negative correlation (r = − 0.61, *P* = 0.04) was determined for Trp, while a moderate positive correlation (r = 0.59, *P* = 0.05) was determined for Gln (Fig. 1). For the remaining AA, correlations with Neff were not significant (*P* ≥ 0.10; Supplementary Table S3).

### Sequencing information

Across quality-filtered, rarefied samples, 2250 bacterial ASV were identified. Rarefaction curves from both sample site and Neff phenotype tended to plateau indicating an adequate sequencing depth for bacterial community analyses (Supplementary Fig. S1). In addition, the Good’s coverage index estimated that at least 97% of the bacterial community was characterized across samples. Thus, analyses in this study accurately evaluated dominant members of the bacterial populations in the rumen and feces from low and high Neff cows.

### Sample site

**Figure 2:**
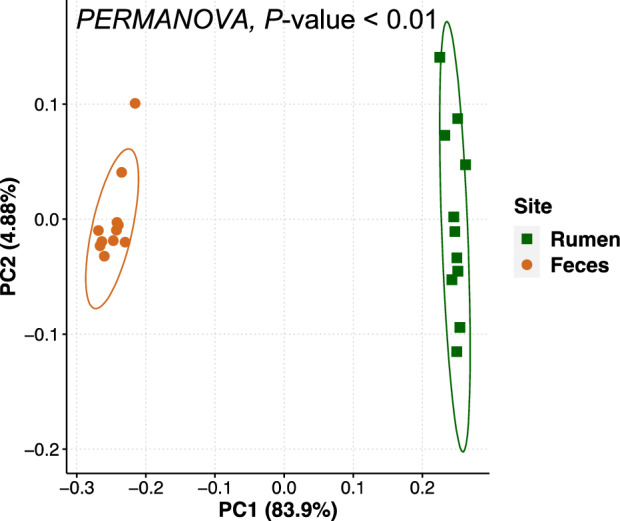
Principal coordinate analysis displaying clustering of bacterial communities by sample site. Beta diversity analysis based on the weighted UniFrac distances. Ellipses represent the 95% confidence interval.

The bacterial communities differed by sample site (*P* ≤ 0.01) which was clearly visualized in the PCoA plot (Fig. 2). In the rumen, *Bacteroidetes*, *Proteobacteria*, and *Firmicutes* were the most abundant phyla and accounted for 74.3% of the total quality-filtered reads. Other predominant phyla were *Spirochaetota* (8.4%), *Verrucomicrobiota* (6.2%), *Fibrobacterota* (6.0%), *Cyanobacteria* (2.7%) and *Patescibacteria* (1.3%). In the fecal samples, the most abundant bacterial phyla were *Bacteroidetes* and *Firmicutes*, which accounted for 88.3% of the total quality-filtered reads. Other predominant phyla in the fecal samples were *Spirochaetota* (5.2%), *Proteobacteria* (2.9%), and *Actinobacteriota* (1.5%). The taxonomic classifications of the rumen and fecal bacterial communities across ranks are shown in Supplementary Fig. S2.

### Diversity analysis

#### α-Diversity

The alpha metrics used to describe the bacterial community richness (observed ASV’s), evenness (Pielou’s index) and diversity (Shannon index) from the rumen and feces in high and low Neff cows are presented in Table 3. In the rumen bacteriome, high Neff cows presented a greater richness than low Neff cows (*P* = 0.04). However, alpha metrics for evenness (*P* = 0.86), and diversity (*P* = 0.47) were similar between both groups. In the fecal bacteriome, alpha metrics for richness (*P* = 0.72), evenness (*P* = 0.99), and diversity (*P* = 0.72) were similar.

**Table 3:** Alpha diversity metrics of the rumen and fecal bacterial communities in low and high N efficient Holstein cows.

Metrics^1^	Site	Phenotype		*P*-value
		Low Neff (n = 6)	High Neff (n = 5)	
Observed ASV’s	Rumen	640.5 ± 17.28	706 ± 21.49	0.04
	Feces	515.5 ± 17.88	524.6 ± 23.86	0.72
Evenness	Rumen	0.80 ± 0.01	0.81 ± 0.02	0.86
	Feces	0.84 ± 0.01	0.84 ± 0.01	0.99
Shannon	Rumen	7.47 ± 0.11	7.65 ± 0.16	0.47
	Feces	7.55 ± 0.10	7.57 ± 0.09	0.72

Neff N efficiency.

^1^Values expressed as mean ± SEM.

#### β-Diversity

**Figure 3:**
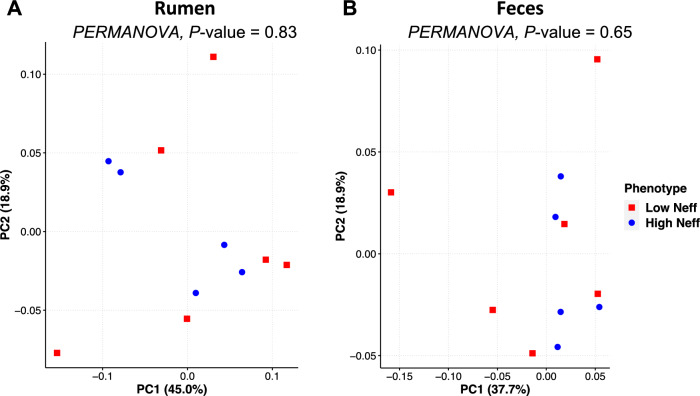
Principal coordinate analysis displaying no clustering of bacterial communities by nitrogen efficiency phenotype from the (**A**) rumen and (**B**) feces of Holstein cows. Beta diversity analysis based on the weighted UniFrac distances.

**Figure 4:**
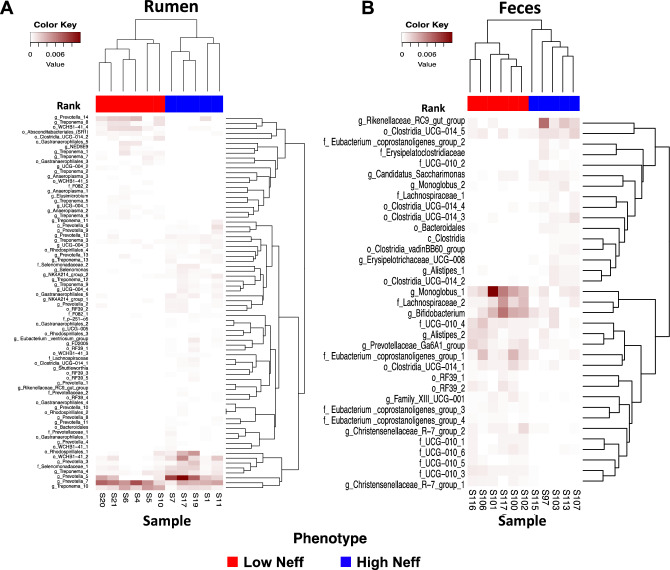
Differentially abundant ASV from the (**A**) rumen and (**B**) fecal bacterial communities in low and high nitrogen efficient Holstein cows. Heatmaps generated using R software v3.6.1.

The phylogenetic composition of the bacterial communities in both the rumen and feces were similar between Neff groups (*P* ≥ 0.65) and this was reflected by no clear grouping of samples in the PCoA plot (Fig. 3). At the ASV-level, 76 ruminal and 35 fecal differentially abundant ASV were identified using LEfSe between low and high Neff cows (Fig. 4). Hierarchical clustering showed distinct groups of differentially abundant ASV between Neff phenotypes in both the rumen and feces. In the rumen, differentially abundant ASV mainly belonged to the genera *Prevotella* (14 ASV) and *Treponema* (13 ASV); whereas in the feces, differentially abundant ASV mainly belonged to the order Clostridia UCG-014 (5 ASV) and to the families *UCG-010* (6 ASV) and *Eubacterium coprostanoligenes *group (4 ASV).

### Correlations between differentially abundant bacterial ASV and Neff

**Figure 5:**
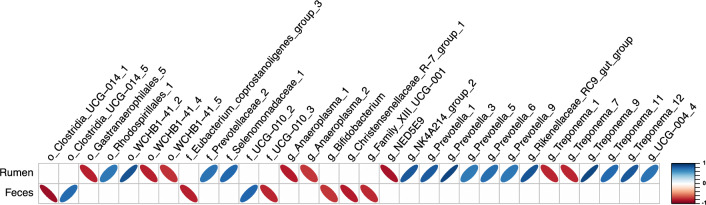
Correlation analysis showing strong associations between differentially abundant bacterial ASV from the rumen and feces and nitrogen efficiency in Holstein cows. Correlation coefficients between 0.71 and 1.00 (− 0.71 and − 1.00) and taxonomy at the highest classified rank are shown.

Correlations were performed to evaluate the relationships between the relative abundance of differentially abundant bacterial ASV from the rumen and feces and Neff (Fig. 5). In the rumen, ASV from the order *Rhodospirillales*, the families *Prevotellaceae* and *Selenomonadaceae*, and the genera *NK4A214 group*, *Prevotella*, *Rikenellaceae RC9 gut group*, and *UCG-004* had strong positive correlations with Neff, whereas ASV from the order *Gastranaerophilales* and the genera *Anaeroplasma* and *NED5E9* had strong negative correlations with Neff. ASV belonging to the order *WCHB1* and the genus *Treponema* had both positive and negative strong correlations with Neff. Moderate correlations were also identified between 24 ruminal ASV and Neff where ASV from the genus *Prevotella* had positive correlations and ASV from the genus *Treponema* mainly had negative correlations (Supplementary Table S4). In feces, 8 differentially abundant ASV were identified to have strong correlations with Neff and the majority (6 ASV) had negative associations and belonged to the order *Clostridia UCG-014*, the families *Eubacterium coprostanoligenes group* and *UCG-010*, and the genera *Bifidobacterium*, *Christensenellaceae R-7 group*, and *Family XIII UCG-001*. In addition, 9 ASV had moderate positive and 6 ASV had moderate negative correlations with Neff (Supplementary Table S5).

## Discussion

In lactating cows, N intake is a main factor affecting the partition of N between milk and manure^5,18^. As N intake increases, there is decrease in N efficiency^18^ and consequently, manure N increases^19,20^. In the current study, Neff groups had a similar N intake, but N partitioned towards milk protein was greater in high Neff compared to low Neff cows. This response was driven by milk yield since milk protein concentration was similar between groups. Similar observations were reported in a study under commercial conditions where herds averaging 30% Neff had higher milk yield and similar milk composition compared to herds averaging 22% Neff^8^. For manure N, high Neff cows excreted less urinary and fecal N per kg of milk than low Neff cows. Nitrogen excreted in manure represents an economic loss and an environmental burden, particularly urinary N which is rapidly lost through volatilization^21,22^.

Rumen microbial fermentation contributes to feedstuff degradation in ruminants. This process provides microbial CP and VFA which are the main sources of AA and energy to the animal, respectively^11,23^. Moreover, the rumen fermentation profile can impact production responses in dairy cows^24^. In ruminant nutrition, the portion of dietary CP that is degraded by rumen microbes is defined as rumen-degradable, whereas the portion that bypasses the rumen is defined as rumen-undegradable. Across feeds, rumen-degradable protein ranges from 30 to 85% and its degradation by rumen microbes yields peptides, AA, and NH_3_ that are used for microbial CP synthesis^10,25^. However, degraded CP not incorporated into microbial CP is converted to NH_3_, absorbed through the rumen wall and eventually disposed as urea in the urine. Given the importance of rumen microbial fermentation on N metabolism, the rumen fermentation profiles between Neff groups were evaluated. Similar rumen environments were observed between high and low Neff groups. In contrast, a study using goats found lower concentrations of NH_3_ and propionic acid in the high Neff group compared to the low Neff group and associated these responses with increased microbial CP synthesis^26^. Discrepancies between studies can potentially be attributed to differences in ruminant species and diet^27,28^. In general terms, plasma concentration of AA reflects the interplay between AA pool inputs and outputs. Meta-analytic research suggests a linear relationship between the predicted individual duodenal EAA flows and their respective plasma concentration in lactating dairy cows^29^. We observed plasma concentration of Gln to be greater in high Neff compared to low Neff cows and a moderate positive correlation between plasma Gln and Neff. Factors driving the difference in plasma Gln were not clear since supply was most likely similar due to similar DMI between Neff groups and Gln generation from the urea cycle might not be a contributor due to similar plasma urea between Neff groups^30^. We also observed a moderate negative correlation between plasma Trp and Neff and this potentially might reflect imbalances of AA as Trp supply increases.

Studies have shown that the microbial community structure in the rumen and lower gut is relevant to animal productivity^12,17^. Commonly, no differences are observed between extreme phenotypes at the community level but at particular taxonomic levels^31,32^. In the present study, beta diversity was similar between Neff groups, but differentially abundant ASV were identified. In the rumen, *Prevotella* is an abundant and core genus^33,34^ and in this study the relative abundances of the majority of the differentially abundant ASV belonging to *Prevotella* were greater in high Neff compared to low Neff cows. In addition, these *Prevotella* species presented strong positive correlations with Neff. Regarding rumen N metabolism, *Prevotella* species were correlated to branched chain AA biosynthesis in high milk protein yield cows suggesting greater microbial CP synthesis^35^. Also, *Prevotella ruminicola* and microbial CP concentration had been reported to be greater in high Neff compared to low Neff goats^26^. In the current study, ASV belonging to the *Treponema* genus were also identified to differ between Neff groups. The *Treponema* genus is another member of the core group of the rumen microbiome^36^ and species of this genus contribute to the ruminal fatty acid pool^37^. Mixed results were observed for microbes from the *Treponema* genus as around half of the differentially abundant ASV were greater in the low Neff group and the remainder were greater in the high Neff group. Likewise, 2 strong negative and 3 strong positive correlations were observed in ASV from the *Treponema* genus.

The profile of the main phyla in the hindgut is similar to that of the rumen, but the relative abundances and the microbes within these groups vary considerably^38^. Previous studies evaluating the bacterial profile from the hindgut in cattle have shown differences in the abundance of community members between contrasting feed efficiency animals and have also reported associations with productive traits such as milk protein^17,39^. The latter suggests that it is relevant to identify bacterial groups across the different sections of the gastrointestinal tract that impact nutrient efficiency. In this study, mainly strong negative correlations were identified between fecal bacterial ASV and Neff and in general these ASV belonged to the class *Clostridia*. Microbes within the *Clostridi*a taxon are obligate anaerobes that commonly reside within the gastrointestinal tract of farm animals and can ferment plant polysaccharides^40^. However, multiple species can produce toxins causing enteric diseases^41^. Likewise, a negative strong correlation between the family *Lachnospiraceae* (within the class *Clostridia*) and Neff was reported in the colon of goats^26^.

## Conclusions

In ruminants, the gastrointestinal microbiome has a central role in N metabolism; however, differences in the bacterial communities between divergent Neff groups are unclear. We evaluated the bacterial community profiles of the rumen and hindgut between low and high Neff dairy cows. In both rumen and fecal samples, we identified clusters of differentially abundant bacterial features that were distinct between low and high Neff cows. In the rumen, differentially abundant species from the genus *Prevotella* showed strong positive correlations with Neff; whereas in feces, differentially abundant species from the class *Clostridia* showed strong negative correlations with Neff. We also report a positive correlation between plasma Gln and Neff. In addition to the rumen microbiome, the microbiome harbored in other sections of the gastrointestinal tract can impact the efficiency N is converted into milk protein.

## Methods

### Animals, housing and management

All experimental procedures involving animals were performed under the approval of the Mississippi State University Institutional Animal Care and Use Committee (IACUC #19-581). The study complies with ARRIVE guidelines and all methods were performed in accordance with the relevant guidelines and regulations. A 28-day trial was conducted with 23 mid-lactation (164 ± 37.7 days in milk; mean ± SD) Holstein cows at 4.8 ± 0.8 years of age with 633.5 ± 49.8 kg of body weight (BW), 2.90 ± 0.40 of body condition score and producing 37.7 ± 6.5 kg/day of milk. The first 14-day served as an adaptation period to the Calan Broadbent Feeding System (American Calan Inc., Northwood, NH, USA) to record individual intake. The diet was formulated to meet or exceed the requirements of a lactating cow^11^ and comprised of 50% forage and 50% concentrate (dry matter (DM) basis; Supplementary Table S6). At the end of the adaptation period, cows were able to access their assigned feed bin without assistance and exhibited stable intake and milk production patterns (Supplementary Fig. S3). The subsequent 14-day served as the collection period of samples.

All cows were housed in a free-stall barn within the same pen with a stocking density of 96% and were under the same management protocol during the study. Cows were provided 2 ×/day (0600 and 1800 h) fresh total mixed ration (TMR) for ad libitum consumption and had free access to water troughs. The amount of TMR offered to each cow was adjusted daily to maintain approximately 10% refusals (as is basis) based on individual refusals from the previous day. Intake was calculated as the difference between the amount of feed offered and refused. The cows were milked 2 ×/day (0300 and 1500 h) in a double 8 parallel milking parlor at which the animals were moved as a group. No health complications were recorded, and all cows completed the study. Following the completion of the study, cows were returned to the standard farm management protocols.

### Nitrogen balance

#### Feed and milk composition

TMR and refusals were sampled on days 21, 22, 27 and 28 and stored at − 20 °C. Composited samples of TMR and refusals were sent to Cumberland Valley Analytical Services (Waynesboro, PA) for DM, CP [^42^; method 990.03], neutral detergent fiber (NDF)^43^, and acid detergent fiber (ADF) [^42^; method 973.18] analyses. Individual milk yield was recorded daily throughout the experiment (Westfalia Surge Metatron 12 Milk Meter; GEA Farm Technologies, Oelde, Germany). From day 24 to 26, milk samples were collected from each milking using Waikato milk meters (Waikato Milking Systems, Horotiu, New Zealand) and stored at room temperature in plastic vials with a tablet preservative containing 8 mg bronopol and 0.30 mg natamycin (Broad Spectrum Microtabs II; Advanced Instruments, Norwood, MA) before shipping to Mid-South Dairy Records (Springfield, MO, USA) for true protein and fat content, and somatic cell count (SCC) analyses by Fourier-transform infrared spectroscopy (Bentley FTS/FCM; Bentley Instruments, Chaska, MN, USA). Calculations for N intake and milk N were as follows:

1 $${\text{N}}\;{\text{intake}} = \left[ {\left( {{\text{intake}} \times {\text{diet}}\;{\text{CP}} / 6.25} \right)} \right] - \left[ {{\text{refusals}} \times \left( {{\text{refusals}}\;{\text{CP}} / 6.25} \right)} \right] \times 1000$$

where N intake = g/day, intake and refusals = kg/day, and diet and refusals CP = proportion of CP in the sample.

2 $${\text{Milk}}\;{\text{N}} = [{\text{milk }}\;{\text{yield }} \times ({\text{milk}}\;{\text{true}}\;{\text{ protein}} / 6.38)] \times 1000$$

where Milk N = g/day, milk yield = kg/day, and milk true protein = proportion of true protein in the sample.

#### Fecal and urinary N

To account for diurnal variation, fecal and urine samples from each cow were concomitantly collected on day 26 at 0000, 0900 and 1800 h, on day 27 at 0300, 1200 and 2100 h, and on day 28 at 0600 and 1500 h (n = 8). This sampling scheme allowed to collect samples representing 3-h intervals in a 24-h period. Fecal samples (~ 200 g) were collected via fecal grab and stored in inert polyethylene cups at − 20 °C. Spot urine samples (~ 450 mL) were collected via vulva stimulation and immediately after each collection, an aliquot of 40 mL was acidified to pH < 3 with 3 mL of 10% sulfuric acid (H_2_SO_4_) and stored at − 20 °C. An additional 40 mL aliquot of urine without acid was stored at − 80 °C. Fecal samples were thawed and composited by cow and then samples were air dried at 60 °C for 48 h and subsequently ground through a 2 mm screen using a Wiley mill (Arthur H. Thompson Co., Philadelphia, PA). Ground fecal samples were analyzed for DM, N [^42^; method 2001.11] using a Kjeltec Auto Sampler System 1035 Analyzer (Tecator Inc., Höganäs, Sweden), and NDF [^42^; method 2002.04] and ADF [^42^; method 973.18] using an Ankom 200 Fiber Analyzer (Ankom Technology, Macedon, NY, USA). Acidified urine samples were thawed, composited by cow, and shipped to Cumberland Valley Analytical Services (Waynesboro, PA) for N analysis [^42^; method 990.03]. Non-acidified urine samples were thawed, composited by cow, and analyzed for creatinine concentration using the creatinine colorimetric assay (Cayman Chemical Co., Ann Arbor, MI, USA) according to the manufacturer’s protocol. Urinary creatinine was used as a marker to estimate urine volume as^44^:

3 $${\text{Urine}}\;{\text{volume}} = 29 \times {\text{BW / creatinine }}$$

where Urine volume = L, creatinine excretion = 29 mg/kg of BW, BW = kg, and creatinine = mg/L. Urinary N losses were then calculated as the product of urine volume and urine N concentration (acid adjusted).

Apparent nutrient digestibility was estimated using the nylon bag techniques with indigestible acid detergent fiber (iADF) as the internal marker^45^. Samples of ground TMR and feces (from each cow) were weighed in triplicates into 5 × 10 cm in situ bags with 50 ± 10 μm pores (R510; Ankom Technology, Macedon, NY, USA) and incubated in the rumen of two cannulated cows for 12 days. The canulated cows were under the same experimental diet. After incubation, the in situ bags were immediately placed on ice and then gently rinsed with running tap water to remove attached particles and stop microbial activity. Digested samples were later dried at 60 °C for 48 h and residues were analyzed for iADF following the procedure specified for the fecal grab samples. Apparent nutrient digestibility (%) was calculated as follows:

4 $$100 - \left[ {100 \times \left( {\frac{{\% \; {\text{of }}\;{\text{marker }}\;{\text{in}}\;{\text{ diet}}}}{{\% \;{\text{of }}\;{\text{marker }}\;{\text{in }}\;{\text{feces}}}}} \right) \times \left( {\frac{{\% \; {\text{of }}\;{\text{nutrient}}\;{\text{ in}}\;{\text{ feces}}}}{{\% \;{\text{of }}\;{\text{nutrient }}\;{\text{in}}\;{\text{ diet}}}}} \right)} \right]$$

Fecal N was then calculated as the difference between N intake and digestible N.

For each cow, Neff was calculated as^8,46^:

5 $${\text{N}}\;{\text{efficiency }} = ({\text{milk}}\;{\text{N / N}}\;{\text{intake}}) \times 100$$

where N efficiency = %, milk N = g/day, and N intake = g/day. Cows with 0.5 standard deviation (SD) below and above the Neff mean were classified as low (Neff < mean + 0.5 × SD) and high (Neff > mean + 0.5 × SD) Neff, respectively^26,47^.

### Bacterial community composition and rumen fermentation

#### Rumen and fecal sampling

On day 27, rumen samples were collected at 0500 h (before morning feeding) using a gastroesophageal apparatus that consisted of a reinforced vinyl tube coupled on one end to a metal strainer and on the other end to a 500 mL sterile collection flask that was connected to a vacuum pump (model 1HAB25BM100X; Gast, Benton Harbor, MI, USA). Rumen sampling and preparation of rumen samples for bacterial community profiling were done as described by Paz et al.^48^. Immediately after sampling, pH was measured using a portable pH meter (handheld pH meter, Oakton, Vernon Hills, IL) by direct insertion into an aliquot of 50 mL of rumen fluid. For ammonia (NH_3_) and volatile fatty acids (VFA) analyses, aliquots of rumen fluid (45 mL each) were filtered through 4 layers of cheesecloth and stored at − 20 °C. Following rumen sampling for each cow, approximately 200 g of feces were collected via fecal grab for bacterial community profiling. Fecal samples were placed in sterile plastic containers and stored at − 80 °C until further analysis.

#### DNA extraction, library preparation, and sequencing

Genomic DNA was extracted from rumen and fecal samples as described by De La Guardia-Hidrogo and Paz^49^. Amplified DNA integrity was checked through gel electrophoresis (1% agarose gel) and concentration and purity (260-to-280 nm ratio) were determined using the NanoDrop One Spectrophotometer (Thermo Fisher Scientific, Wilmington, DE, USA). Bacterial amplicon libraries of the V4 region from the 16S rRNA gene were prepared as described by Kozich et al.^50^. Briefly, each 20 μL PCR amplification reaction contained 0.5 μL Terra PCR Direct Polymerase Mix (0.625 Units; Takara Bio, Mountain View, CA, USA), 7.5 μL nuclease-free, sterile water, 10 μL 2 × Terra PCR Direct Buffer (Takara Bio, Mountain View, CA, USA), 1 μL indexed primers (10 μM) and 1 μL DNA (25–70 ng DNA). The cycling conditions included an initial denaturation of 98 °C for 3 min, followed by 25 cycles of 98 °C for 30 s, 55 °C for 30 s, and 68 °C for 45 s, and a final extension of 68 °C for 4 min. The resulting amplicons were normalized (1–2 ng/μL) using the SequalPrep™ Normalization Plate Kit (Invitrogen, Carlsbad, CA, USA) according to the manufacturer’s protocol. Normalized amplicons were pooled (10 μL/sample) and libraries were purified using the MinElute PCR Purification Kit (Qiagen Inc., Valencia, CA, USA) as described by the manufacturer. Finally, libraries were sequenced using the Illumina MiSeq platform (Illumina, San Diego, CA, USA) at the Institute for Genomics, Biocomputing, and Biotechnology (Mississippi State, MS, USA). Raw sequences are available at the NCBI Sequence Read Archive (SRA) under the accession no. SRP400507.

#### Ruminal fermentation

Prior to analysis, samples for NH_3_ and VFA were thawed. For NH_3_ concentration, a portable meter (Orion meter model 290; Thermo Scientific Inc., Waltham, MA, USA) coupled to an NH_3_ ion selective electrode (Orion 9512 ammonia sensing electrode; Thermo Scientific Inc., Waltham, MA, USA) was used. For VFA profile, 1 mL of filtered rumen fluid was mixed with 0.2 mL of an internal standard (2-mg/mL 2-ethyl butyric acid in 25% meta-phosphoric acid (w/v)). Subsequently, samples were centrifuged at 18,000×*g* for 20 min, and the resulting supernatant was analyzed using a 7890A gas chromatography system equipped with a DB-FATWAX Ultra Inert column (30 m × 0.25 mm, 0.25 µm), a 5975C inert XL MSD with triple-axis mass detector, and a 7693 series autosampler (Agilent Technologies, Santa Clara, CA, USA). Ionization was performed in an electron impact mode at 70 eV and a selected ion monitoring mode was used to acquire ion abundance. Volatile fatty acids were quantified by an internal standard calibration with authentic VFA standards.

### Plasma amino acids (AA)

To evaluate plasma AA profiles between Neff groups, blood samples were collected on day 27 at 1800 h via venipuncture of the coccygeal artery. Samples were collected into evacuated tubes containing K_2_EDTA (BD Vacutainer; Becton Dickinson and Co., Franklin Lakes, NJ, USA). Immediately after collection, samples were placed on ice and plasma was separated as described by Paz and Kononoff^51^. Plasma samples were stored at − 20 °C and later shipped to the Agricultural Experimental Station Chemical Laboratories (Columbia, MO, USA) for free plasma AA analysis using a Hitachi L-8900 AA analyzer (Hitachi Ltd., Tokyo, Japan)^52,53^.

### Bioinformatics analysis

Detailed information of the complete bioinformatic analysis containing the metadata file and sequential R markdown files with the scripts and commands used in this study can be found at https://github.com/pazlabgit/Neff_holstein_pipeline. Briefly, demultiplexed sequences were analyzed using QIIME 2 v2020.8.0^54^. The denoising process included an initial quality filtering based on quality scores (q2-quality-filter plugin)^55^ followed by the Deblur workflow^56^. Quality-filtered sequences were clustered into amplicon sequence variants (ASV) and taxonomy was assigned on representative sequences using a Naives Bayes classifier^57^ trained on the Silva database v138^58^. The hierarchical structure of the taxonomic classifications from the bacterial communities was visualized with heat trees using the "metacoder" package^59^ from the R software v3.6.1^60^. Rarefaction curves based on observed ASV and the Good’s coverage index^61^ were used to evaluate the adequacy of the sampling depth, which was established at 5834 quality-filtered reads per sample. Diversity analyses were performed via the q2-diversity plugin with α-diversity metrics determined using observed ASV, Pielou’s evenness^62^, and Shannon–Weiner index^63^ and β-diversity calculated using the weighted UniFrac distances. Visualization of the weighted UniFrac distances was done using the principal coordinate analysis (PCoA) plot.

### Statistical analysis

The statistical analyses were conducted using the R software. Prior to analysis, normality was confirmed using the Shapiro–Wilk test (*P* ≥ 0.13) for dry matter intake (DMI), milk yield, plasma AA, N balance and Neff data. A student *t*-test was used to compare the aforementioned variables between the low and high Neff groups. Analyses for DMI and milk yield and composition were performed on the collection period data when milk samples were taken (3 days). α-diversity indices were compared using the Kruskal–Wallis test and β-diversity was analyzed with a permutational multivariate ANOVA (PERMANOVA) using the adonis function of the "vegan" package. Differentially abundant ASV within the rumen and fecal bacteriomes between Neff phenotypes were identified using the linear discriminant analysis effect size (LEfSe) implemented in the Galaxy server v1.0 (https://huttenhower.sph.harvard.edu/galaxy/) with default settings. Pearson correlation was used to assess the relationship between individual plasma AA and Neff, while the Spearman’s rank correlation was used to assess the relationship between differentially abundant ASV from the rumen and feces and Neff. The *P*-values were adjusted for multiple testing using the false discovery rate (FDR) method. Correlation coefficients between 0.51 and 0.70 (− 0.51 and − 0.70), and between 0.71 and 1.00 (− 0.71 and − 1.00) were defined as moderate and strong, respectively^64^. Statistical significance was declared at *P* ≤ 0.05.

### Ethics approval and consent to participate

All animal procedures were approved by the Mississippi State University Institutional Animal Care and Use Committee (IACUC #19-581).

## Supplementary Information

Supplementary Figure S1.

Supplementary Figure S2.

Supplementary Figure S3.

Supplementary Table S1.

Supplementary Table S2.

Supplementary Table S3.

Supplementary Table S4.

Supplementary Table S5.

Supplementary Table S6.

## Data Availability

Raw sequences are available at the NCBI Sequence Read Archive (SRA) under the accession no. SRP400507. Detailed information of the complete bioinformatic analysis containing the metadata file and sequential R markdown files with the scripts and commands used in this study can be found at https://github.com/pazlabgit/Neff_holstein_pipeline.

## Abbreviations

AA: Amino acids
ADF: Acid detergent fiber
ASV: Amplicon sequence variants
BW: Body weight
CP: Crude protein
DM: Dry matter
DMI: Dry matter intake
EAA: Essential amino acids
H_2_SO_4_: Sulfuric acid
iADF: Indigestible acid detergent fiber
LEfSe: Linear discriminant analysis effect size
NCBI: National center for biotechnology information
NDF: Neutral detergent fiber
NEAA: Non-essential amino acids
Neff: Nitrogen efficiency
NH_3_: Ammonia
PCoA: Principal coordinate analysis
PCR: Polymerase chain reaction
PERMANOVA: Permutational multivariate analysis of variance
QIIME 2: Quantitative insights into microbial ecology 2
SCC: Somatic cell count
SD: Standard deviation
SEM: Standard error of the mean
SRA: Sequence read archive
TEAA: Total essential amino acids
TMR: Totally mixed ration
VFA: Volatile fatty acids
