# Supplementary material for: Bacterial community structure in the rumen and hindgut is associated with nitrogen efficiency in Holstein cows
Source: Sci Rep. 2023 Jul 3;13:10721. doi: 10.1038/s41598-023-37891-7 (PMC10317951; doi:10.1038/s41598-023-37891-7)
Supplement: Supplementary file 1 — Supplementary Figure S1. [file 41598_2023_37891_MOESM1_ESM.pdf]

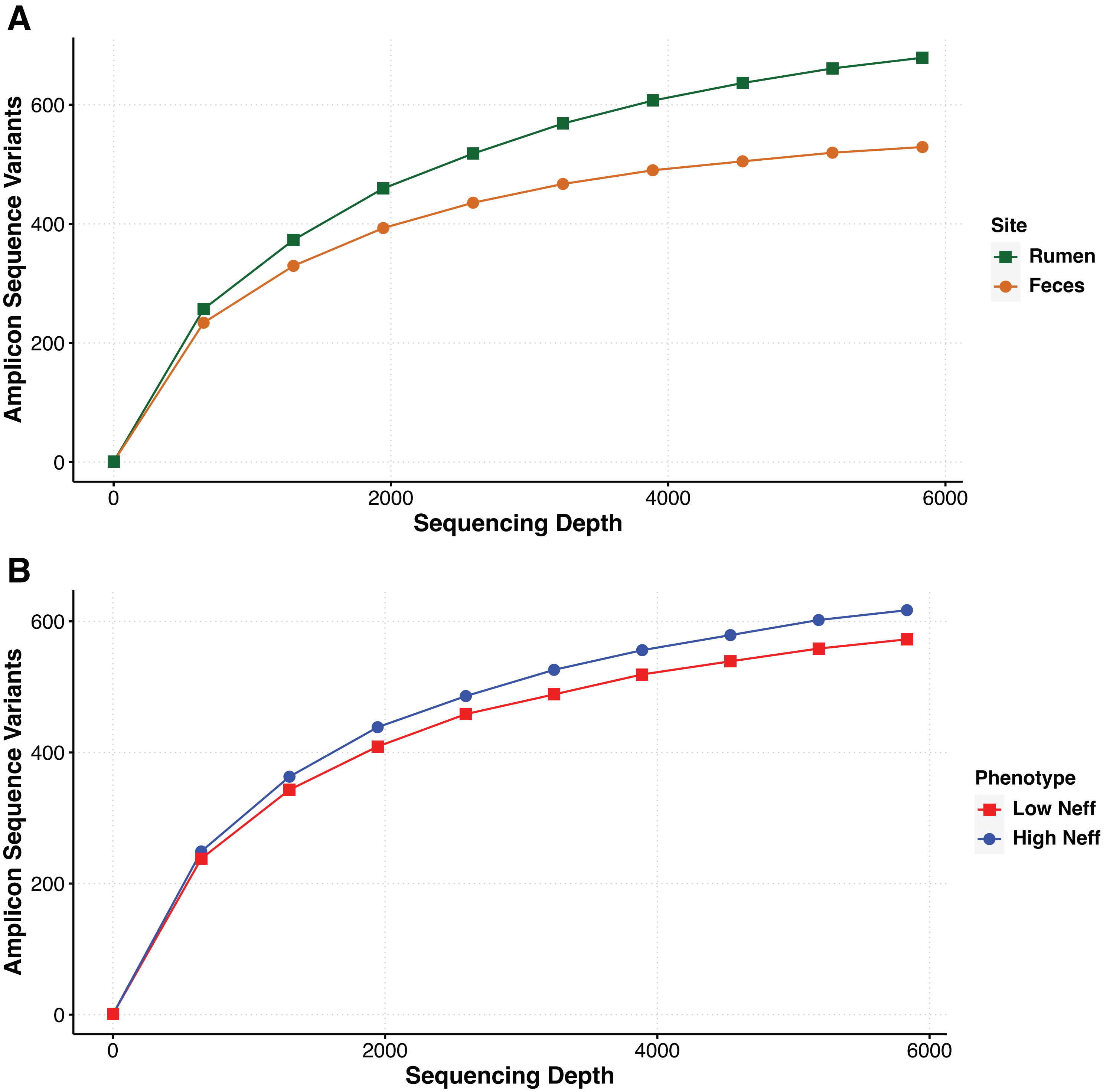

Supplementary Figure S1. Rarefaction curves between observed amplicon sequence variants and sequencing depth. Curves in (**A**) and (**B**) for sample site and nitrogen efficiency phenotype, respectively.
