# Supplementary material for: Bacterial community structure in the rumen and hindgut is associated with nitrogen efficiency in Holstein cows
Source: Sci Rep. 2023 Jul 3;13:10721. doi: 10.1038/s41598-023-37891-7 (PMC10317951; doi:10.1038/s41598-023-37891-7)
Supplement: Supplementary file 2 — Supplementary Figure S2. [file 41598_2023_37891_MOESM2_ESM.pdf]

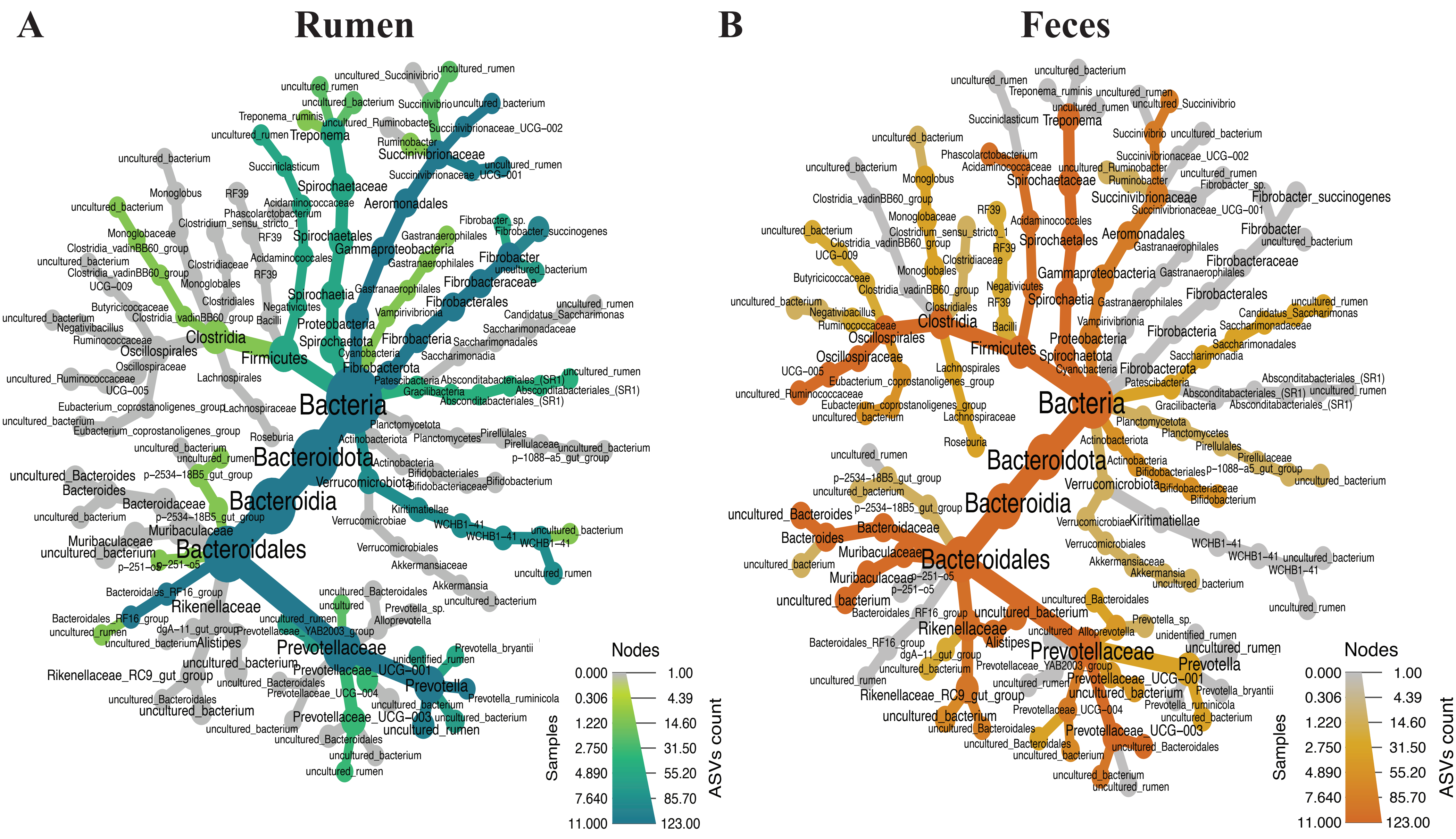

Supplementary Figure S2. Taxonomic composition of the bacterial communities of Holstein cows. (A) Rumen and (B) feces heat trees. Size and color of the nodes (circles) and edges (lines) correspond to the relative abundance of the respective taxonomic levels. Heat trees generated using R software v3.6.1.
