# Supplementary material for: Bacterial community structure in the rumen and hindgut is associated with nitrogen efficiency in Holstein cows
Source: Sci Rep. 2023 Jul 3;13:10721. doi: 10.1038/s41598-023-37891-7 (PMC10317951; doi:10.1038/s41598-023-37891-7)
Supplement: Supplementary file 3 — Supplementary Figure S3. [file 41598_2023_37891_MOESM3_ESM.pdf]

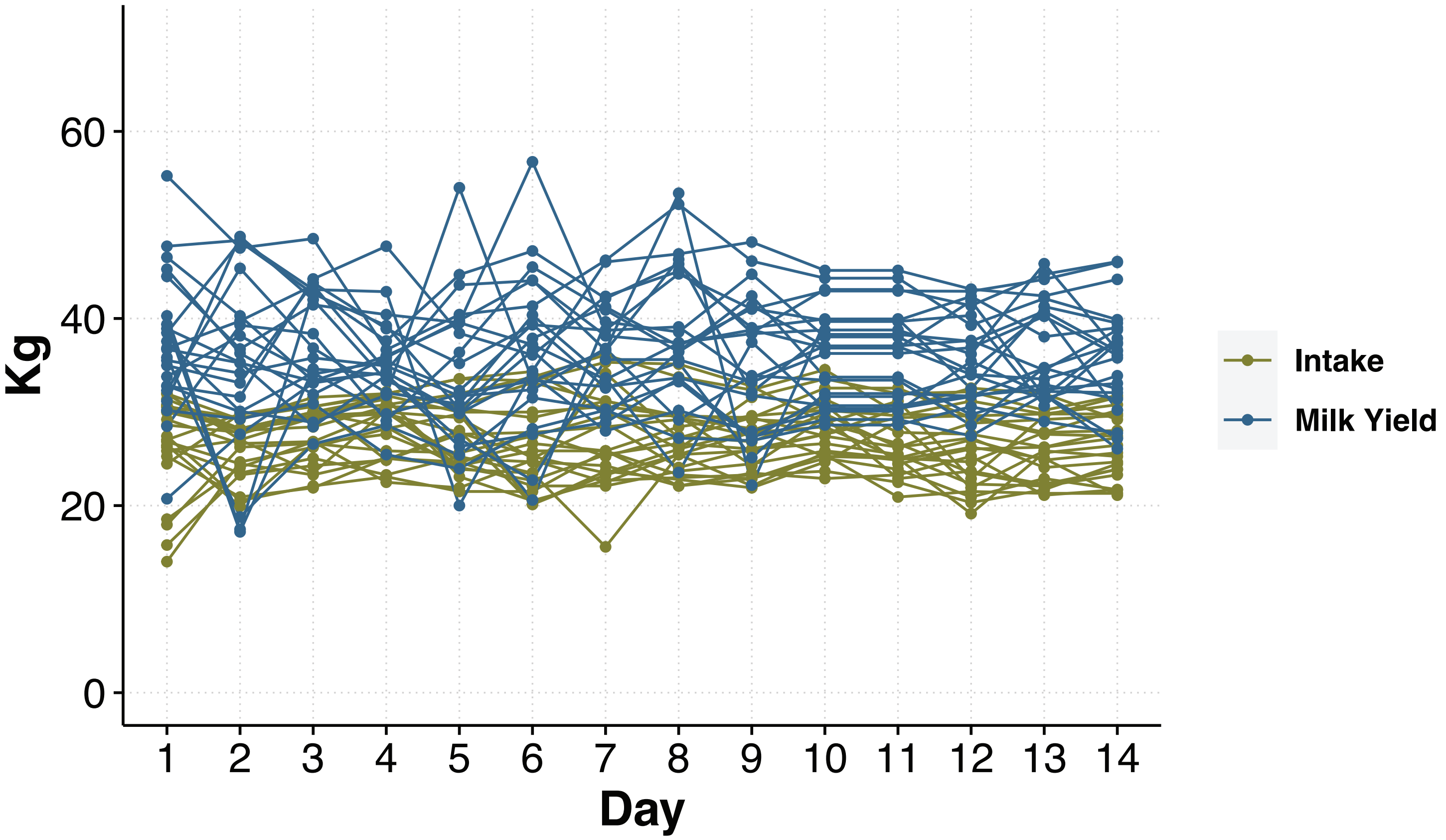

Supplementary Figure S3. Dry matter intake and milk yield patterns during the adaptation period to the Calan Broadbent Feeding System.
