# Supplementary material for: Bacterial community structure in the rumen and hindgut is associated with nitrogen efficiency in Holstein cows
Source: Sci Rep. 2023 Jul 3;13:10721. doi: 10.1038/s41598-023-37891-7 (PMC10317951; doi:10.1038/s41598-023-37891-7)
Supplement: Supplementary file 4 — Supplementary Table S1. [file 41598_2023_37891_MOESM4_ESM.pdf]

Supplementary Table S1. Nitrogen efficiency of Holstein cows categorized within low and high groups.

Cow ID	Low Neff	Cow ID	High Neff	*P*-value
414	20.7	438	28.2	
420	21.9	536	28.4	
422	22.4	377	29.2	
539	22.8	474	31.8	
469	24.1	509	34.1	
541	25.0			
**Mean**	**22.8 ± 0.83**	**Mean**	**30.3 ± 0.91**	**< 0.01**
