# Supplementary material for: Bacterial community structure in the rumen and hindgut is associated with nitrogen efficiency in Holstein cows
Source: Sci Rep. 2023 Jul 3;13:10721. doi: 10.1038/s41598-023-37891-7 (PMC10317951; doi:10.1038/s41598-023-37891-7)
Supplement: Supplementary file 5 — Supplementary Table S2. [file 41598_2023_37891_MOESM5_ESM.pdf]

Supplementary Table S2. Plasma free amino acids profile in low and high nitrogen efficient Holstein cows.

	Phenotype[1]		
Item[2]	Low Neff (n = 6)	High Neff (n = 5)	*P*-value
EAA[3], µg/mL			
Arg	14.2 ± 1.50	12.6 ± 1.64	0.46
His	10.31 ± 0.90	9.13 ± 0.99	0.39
Ile	17.7 ± 1.36	14.3 ± 1.49	0.11
Leu	28.8± 2.14	23.4 ± 2.34	0.12
Lys	13.5 ± 1.67	11.1 ± 1.83	0.34
Met	3.25 ± 0.47	2.73 ± 0.512	0.44
Phe	10.36 ± 0.75	7.99 ± 0.83	0.06
Thr	11.9 ± 1.37	12.5 ± 1.50	0.75
Trp	7.57 ± 0.47	6.57 ± 0.51	0.17
Val	36.5 ± 2.45	29.5 ± 2.68	0.08
TEAA[4], µg/mL	154.1 ± 10.6	129.9 ± 11.6	0.14
NEAA[5], µg/mL			
Ala	21.7 ± 2.25	23.3 ± 2.47	0.63
Asp	6.64 ± 0.86	6.98 ± 0.94	0.78
Cys	5.63 ± 0.33	4.88 ± 0.36	0.15
Gln	29.6 ± 1.58	36.6 ± 1.73	0.02
Gly	20.8 ± 2.29	25.4 ± 2.51	0.18
Pro	11.3 ± 1.19	11.4 ± 1.30	0.93
Ser	8.86 ± 1.09	9.04 ± 1.19	0.91
Tyr	11.75 ± 1.14	9.44 ± 1.25	0.19
Urea, µg/mL	311 ± 15.7	309 ± 17.2	0.93

[1]Neff = N efficiency
[2]Values expressed as mean ± SEM
[3]EAA = essential amino acids
[4]TEAA = total essential amino acids (sum of Arg, His, Ile, Leu, Lys, Met, Phe, Thr, Trp, Val)
[5]NEAA = non-essential amino acids
