# Supplementary material for: Bacterial community structure in the rumen and hindgut is associated with nitrogen efficiency in Holstein cows
Source: Sci Rep. 2023 Jul 3;13:10721. doi: 10.1038/s41598-023-37891-7 (PMC10317951; doi:10.1038/s41598-023-37891-7)
Supplement: Supplementary file 6 — Supplementary Table S3. [file 41598_2023_37891_MOESM6_ESM.pdf]

Supplementary Table S3. Correlations between plasma free amino acids and nitrogen efficiency in Holstein cows.

Item	N efficiency (*ρ*)	*P*-value
EAA[1], µg/mL		
Arg	-0.23	0.49
His	-0.38	0.25
Ile	-0.46	0.15
Leu	-0.41	0.21
Lys	-0.38	0.25
Met	-0.16	0.63
Phe	-0.52	0.10
Thr	0.03	0.94
Trp	-0.61	0.04
Val	-0.46	0.15
TEAA[2], µg/mL	-0.45	0.17
NEAA[3], µg/mL		
Ala	0.20	0.55
Asp	0.07	0.84
Cys	-0.38	0.26
Gln	0.59	0.05
Gly	0.43	0.19
Pro	0.08	0.81
Ser	0.12	0.73
Tyr	-0.43	0.19

[1]EAA = essential amino acids
[2]TEAA = total essential amino acids (sum of Arg, His, Ile, Leu, Lys, Met, Phe, Thr, Trp, Val)
[3]NEAA = non-essential amino acids
