# Supplementary material for: Bacterial community structure in the rumen and hindgut is associated with nitrogen efficiency in Holstein cows
Source: Sci Rep. 2023 Jul 3;13:10721. doi: 10.1038/s41598-023-37891-7 (PMC10317951; doi:10.1038/s41598-023-37891-7)
Supplement: Supplementary file 7 — Supplementary Table S4. [file 41598_2023_37891_MOESM7_ESM.pdf]

Supplementary Table S4. Ruminal differentially abundant ASV exhibiting significant correlations with N efficiency in Holstein cows.

Bacterial ASV	Taxonomy	***ρ***	*P*-value
Strong correlations			
13b7e70051e0663052d52f962ef40e1a	o_Gastranaerophilales_5	-0.78	< 0.01
d57112cbfedf6e7db7d80e885eedd3a8	o_Rhodospirillales_1	0.72	0.01
6cd36c95206f3c144679670411420e4e	o_WCHB1-41_2	0.87	< 0.01
a963c2595bdfe837b9f7c6a5667c345f	o_WCHB1-41_4	-0.78	< 0.01
a7f847517e37eb23c63d73f36b2ccfc2	o_WCHB1-41_5	-0.72	0.01
cd8f43429ec8ac6ea86d825cf389a146	f_Prevotellaceae_2	0.73	0.01
df8bd393251b799a607b605886d2433a	f_Selenomonadaceae_1	0.79	< 0.01
642a079967fb2ac33b7a47589143cef9	g_Anaeroplasma_1	-0.79	< 0.01
c8da393dafabaf9ebc14c6c1c0ab0251	g_Anaeroplasma_2	-0.71	0.02
87e7c3cbd0dcc604561471a77905ae9e	g_NED5E9	-0.83	< 0.01
58843e0288d9096e3b2a473146da085b	g_NK4A214_group_2	0.85	< 0.01
f58a692668d9c575fcd2a04f9924fcff	g_Prevotella_1	0.84	< 0.01
52dd2e13f02b87fc59c92b11b0f682b4	g_Prevotella_3	0.92	< 0.01
b6db20730434475b02dd5889fbfd845f	g_Prevotella_5	0.71	0.02
c4fa2e2674a35f5574cbfd63cc08dd71	g_Prevotella_6	0.76	0.01
728cd79a0775b862123442874251 83f0	g_Prevotella_9	0.71	0.01
c464eac66fc80a6cab5fc63581b26f11	g_Rikenellaceae_RC9_gut_group	0.85	< 0.01
c636c2dd3551c4bc40a22f3d38870c56	g_Treponema_1	-0.75	0.01
8149bed0f651af0b8add6d2201c0929c	g_Treponema_7	0.77	0.01
a3575915d0dcad7e6b130222efefb7a6	g_Treponema_9	0.84	< 0.01
59cc67d19e26fef0854c8d4876fae9bd	g_Treponema_11	-0.76	0.01
a198df70078d612198000615d6dc5652	g_Treponema_12	0.90	< 0.01
ed1f4aeb9f049810fc3ee993bad2548d	g_UCG-004_4	0.73	0.01
Moderate correlations			
c44acb300d95adbe026d9d70244e1e01	o_Clostridia_UCG-014_1	0.65	0.03
ae5b637a9462947aad715b33c0dcf0b0	o_Gastranaerophilales_3	-0.61	0.05

8725409d5888523575c5f53b40b73941	o_Gastranaerophilales_4	0.67	0.02
f1b46bb10759b9ed8bb96f7e7f4f2949	o_RF39_4	0.68	0.02
efa243155ab25618eca3b83762710bd0	o_RF39_5	0.67	0.02
ee87a75807cc38e74d10d7d825dacbb8	o_Rhodospirillales_2	0.67	0.02
68a8ecbc9d563da58e9365cdb84d2bd4	o_Rhodospirillales_4	0.65	0.03
3bf62a415381ded69aeaad2548ec9aeb	o_WCHB1-41_1	0.63	0.04
baf6c7d7ee234bf94e78e8a940b4c90c	f_Selenomonadaceae_2	0.70	0.02
ecfea1837c55c7587f33d75187f235f5	g_Anaeroplasma_3	-0.64	0.03
92e53d1a69fb186a117502da69f17d96	g_Prevotella_10	0.67	0.02
7908fab07437eeda542ac091e1fa1871	g_Prevotella_11	0.65	0.03
756976b9893136b2f01192bfa01eb3c8	g_Prevotella_12	0.66	0.03
b3c22df3d7615c43c5710e425a5c90ab	g_Prevotella_2	0.68	0.02
a45d73662c1703043d9eb1c7fda4a19a	g_Prevotella_4	0.68	0.02
2d76a91c290360b6e39072b394c88edd	g_Prevotella_8	0.62	0.04
2fda6ab284ccb1b32078f3ab9a4ed9b2	g_Selenomonas	0.67	0.02
c54d5ba2dce8f98ddf977890c4879952	g_Treponema_10	-0.66	0.03
149409862be7d2169149a9bc9e21fa07	g_Treponema_2	-0.65	0.03
226701542c86d92de97542ae25f59414	g_Treponema_3	0.63	0.04
f1dc13db6fbadb425edbe15bacc2e34a	g_Treponema_6	-0.61	0.05
3e0c9e689d5b6538c1b20cc826c7a73c	g_Treponema_8	-0.67	0.02
56b55fb405990ff910b9015ff08c1d66	g_UCG-004_2	-0.61	0.05
1f162461d6bb086cc78e4edb3d5f740b	g_UCG-005	0.62	0.04
