# Supplementary material for: Bacterial community structure in the rumen and hindgut is associated with nitrogen efficiency in Holstein cows
Source: Sci Rep. 2023 Jul 3;13:10721. doi: 10.1038/s41598-023-37891-7 (PMC10317951; doi:10.1038/s41598-023-37891-7)
Supplement: Supplementary file 8 — Supplementary Table S5. [file 41598_2023_37891_MOESM8_ESM.pdf]

Supplementary Table S5. Fecal differentially abundant ASV exhibiting significant correlations with N efficiency in Holstein cows.

Bacterial ASV	Taxonomy	***ρ***	*P*-value
Strong correlations			
a77418db84c35cdba6e1254aeaa534f1	o_Clostridia_UCG-014_1	-0.86	< 0.01
c2cff75edcac2f18056a41a735694b2e	o_Clostridia_UCG-014_5	0.78	< 0.01
a46eaa77a656e7327582b59efe77b66c	f_Eubacterium coprostanoligenes group_3	-0.78	< 0.01
5f9c74c4553fe8428f316027ae296e67	f_UCG-010_2	0.80	< 0.01
9ac2780339e743a62ef72b60d348878f	f_UCG-010_3	-0.76	0.01
ec14a68bf134b5c86ac231307bfde98b	g_Bifidobacterium	-0.71	0.01
659bc8e39173706faac19baaab085725	g_Christensenellaceae R-7 group_1	-0.79	< 0.01
6eac8a15a307b2e12f5f62f60e47423a	g_Family_XIII_UCG-001	-0.74	0.01
Moderate correlations			
f698e96ae7e9b7ba774dfa06ceeb780e	c_Clostridia	0.65	0.03
494588d6ae286b9b813ea1ccb2da2253	o_Bacteroidales	0.66	0.03
39d583d93c94260ab48e54ae2aa66037	o_Clostridia_vadinBB60_group	0.67	0.02
7a8f38ac3f190f69763f94b3e1b8099e	f_Eubacterium coprostanoligenes group_1	-0.62	0.04
473c3452c4358403cac1a1a3c9f0636b	f_Eubacterium coprostanoligenes group_2	0.60	0.05
3e1abb88a017dc6a6cfe0cf0a88cb4f4	f_UCG-010_5	-0.63	0.04
6b26d47b3210ad11d7b8e61facda68bb	g_Alistipes_1	0.67	0.02
ef13338902dec10bdf6d6b85d25d8005	g_Alistipes_2	-0.59	0.05
2667e29bfc41a7342c6112a78835bead	g_Candidatus_Saccharimonas	0.62	0.04

06438d2facf851955509149395d620d9	g_Christensenellaceae_R-7_group_2	-0.63	0.04
15750cbcc14a283c0801c943dc2044a4	g_ErysipelotrichaceaeUCG-008	0.66	0.03
da46dc8efca41f6859f76ceedbca0ba8	g_Monoglobus_1	-0.69	0.02
12b13eb93378a4203d43196eb0cd068d	g_Monoglobus_2	0.65	0.03
18ec6531a935d2bcb2a0fdaa1d5e73fb	g_Prevotellaceae_Ga6A1group	-0.63	0.04
bb0fb06da3a5a5e09cb71ea49ad49fd0	g__Rikenellaceae_RC9_gut_group	0.64	0.03
