# Supplementary material for: Bacterial community structure in the rumen and hindgut is associated with nitrogen efficiency in Holstein cows
Source: Sci Rep. 2023 Jul 3;13:10721. doi: 10.1038/s41598-023-37891-7 (PMC10317951; doi:10.1038/s41598-023-37891-7)
Supplement: Supplementary file 9 — Supplementary Table S6. [file 41598_2023_37891_MOESM9_ESM.pdf]

Supplementary Table S6. Ingredient and chemical composition of the diet.

Item	% DM
Ingredient	
Corn silage	38.4
Millet Baleage	9.60
Grass hay	2.11
Ground corn	22.4
Soybean meal	11.6
Distillers dried grains with solubles	8.30
Amino acid supplement[1]	1.9
Rumen inert fat[2]	1.9
Minerals and vitamins mix[3]	3.9
Assayed composition	
DM, % as fed	44.0
CP	16.0
NDF	34.1
NEL (Mcal/kg)	1.65

[1]Pro-Team70 (Perdue Agribusiness, Salisbury, MD).

[2]Energy Booster 100 (Milk Specialties Global, Eden Prairie, MN).

[3]Contained 11.2% Ca, 0.4% P, 4.5% Mg, 4.9% K, 12.5% Na, 7.4% Cl, 1.1% S, 1918 mg of Mn/kg, 304 mg of Cu/kg, 1991 mg of Zn/kg, 20 mg of I/kg, 14 mg of Co/kg, 7 mg of Se/kg, 63,960 IU of vitamin A/kg, 16964 IU of vitamin D/kg, and 379 IU of vitamin E/kg.
